# A preliminary simulation-based qualitative study of healthcare students’ experiences of interprofessional primary care scenarios

**DOI:** 10.1186/s41077-022-00204-5

**Published:** 2022-03-21

**Authors:** Lene Lunde, Anne Moen, Rune B. Jakobsen, Britta Møller, Elin O. Rosvold, Anja M. Brænd

**Affiliations:** 1 Department of Nursing Science, Institute of Health and Society, Faculty of Medicine, University of Oslo, Oslo, Norway; 2 Department of Health Management and Health Economics, Institute of Health and Society, Faculty of Medicine, University of Oslo, Oslo, Norway; 3 Department of Communication and Psychology, Humanistic Faculty, Aalborg University, Aalborg, Denmark; 4 Department of General Practice, Institute of Health and Society, Faculty of Medicine, University of Oslo, Oslo, Norway

**Keywords:** Simulation, Interprofessional, Primary care, Healthcare students, Sub-acute scenarios, Focus group

## Abstract

**Background:**

Introducing interprofessional education (IPE) in healthcare curricula can prepare students for healthcare practices that have become increasingly complex. The use of simulation is promoted to support IPE. This study explores healthcare students’ experiences of participating in common, sub-acute patient scenarios that routinely occur in clinical practice in primary care. More specifically, it looks at how sub-acute patient scenarios from primary care can help develop interprofessional collaborative competence.

**Methods:**

Medical students (*N* = 10), master’s students in advanced geriatric nursing (*N* = 8) and bachelor’s students in nursing (*N* = 9) participated in the simulations. The students were in their last or second-to-last year of education. We conducted five semi-structured focus group interviews with the participants’ directly after the simulation training to elicit experiences related to the scenarios, the simulation and interprofessional collaboration. The transcripts were analysed using systematic text condensation. To supplement the focus group interviews, the students also completed the interprofessional collaborative competency attainment survey (ICCAS), which measures the students’ self-assessed interprofessional competence.

**Results:**

Three main themes emerged from the analysis of the focus group interviews: *realism*, *uncertainty* and *reflection*. The students emphasised the importance of authentic and recognisable scenarios. They said the vague and unspecific patient symptoms created uncertainty in the situation, making it difficult to understand the patient’s diagnosis. Despite that uncertainty, they described the experience as positive. Further, the students expressed that the simulation increased their confidence in interprofessional collaboration and prepared them for future work. The results from the ICCAS questionnaire showed that the students reported a subjective positive change in their interprofessional competence after participating in the scenarios.

**Conclusions:**

This study showed that simulation-based IPE with sub-acute primary care scenarios contributes to develop interprofessional collaborative competence in healthcare education. Sub-acute scenarios can supplement the more common approaches with acute care scenarios and aid in developing the collaborative competence required to work in healthcare teams.

**Supplementary Information:**

The online version contains supplementary material available at 10.1186/s41077-022-00204-5.

## Background

Interprofessional education (IPE) is a critical component in healthcare curricula and can help prepare students for healthcare practices that have become increasingly complex [1, 2]. However, there is no widespread educational consensus on how to conduct IPE so that it better prepares students to collaborate across healthcare disciplines. Traditionally, healthcare students are educated in professional silos [3, 4]. As such, traditional teaching does not promote students’ interactions with other healthcare professions. It is a common assumption that students’ exposure to, and involvement in, teamwork occurs naturally in clinical practice and, consequently, prepares the students for working in interprofessional teams. However, there is no guarantee that without purposeful organisation, students will experience exemplary teamwork or even collaborate with other healthcare professionals or students during clinical practice [5]. As a result, healthcare education needs to find approaches that expose students to interprofessional collaboration (IPC).

The use of simulation provides learning experiences where the students are placed in realistic and safe clinical situations [6]. A growing body of research promotes simulation as an educational strategy to support IPE in healthcare education [7–10]. Most simulation-based IPE experiences have focused on life-threatening, time-critical acute-care scenarios [11–14]. While it is important for healthcare students to learn and practice how to respond to severe, acute care scenarios, everyday clinical situations are rife with IPC. Shorter hospital stays and an increased emphasis on home care and ageing in place suggest that more patients with increasingly complex needs will require treatment in a primary care setting [15]. In contrast to most acute care algorithm-based scenarios, sub-acute patient scenarios in primary care provide the students with more time to solve a problem, but the actual clinical situation may be more complex. Introducing simulation training of scenarios typical of primary care can therefore contribute to the students’ learning experiences of IPC.

With this in mind, we developed simulation-based IPE with sub-acute patient scenarios that would commonly occur in clinical practice. The main aim of this article is to explore healthcare students’ experiences of participating in the sub-acute patient scenarios. Specifically, we aimed to understand how the use of sub-acute patient scenarios from primary care could support the development of interprofessional collaborative competence.

## Methods

### Research design and setting

We conducted a qualitative study, using focus group (FG) interviews to capture experiences from students participating in IPE simulation sessions. This is part of an exploratory study exploring different aspects of simulation as a strategy for training healthcare students in IPC in future curricula development. We developed scenarios comprised of sub-acute situations from primary care. The IPE simulation we developed is not yet implemented in our healthcare curricula. FG interviews were considered well suited to elicit experiences and views from the participants and encourage group dialogue after participating in a joint experience such as simulation-based IPE [16]. A series of questions addressing experiences related to the scenarios, the simulation and IPC acted as a guide for the semi-structured interviews. The participants were encouraged to elaborate on topics they considered relevant and important (Additional file 1). In addition, to supplement the FG interviews, the students completed the Norwegian version of the interprofessional collaborative competency attainment survey (ICCAS). ICCAS captures the students’ self-assessment of their interprofessional competence and is validated across various settings and countries, including Norway [17–19].

**Fig. 1:**
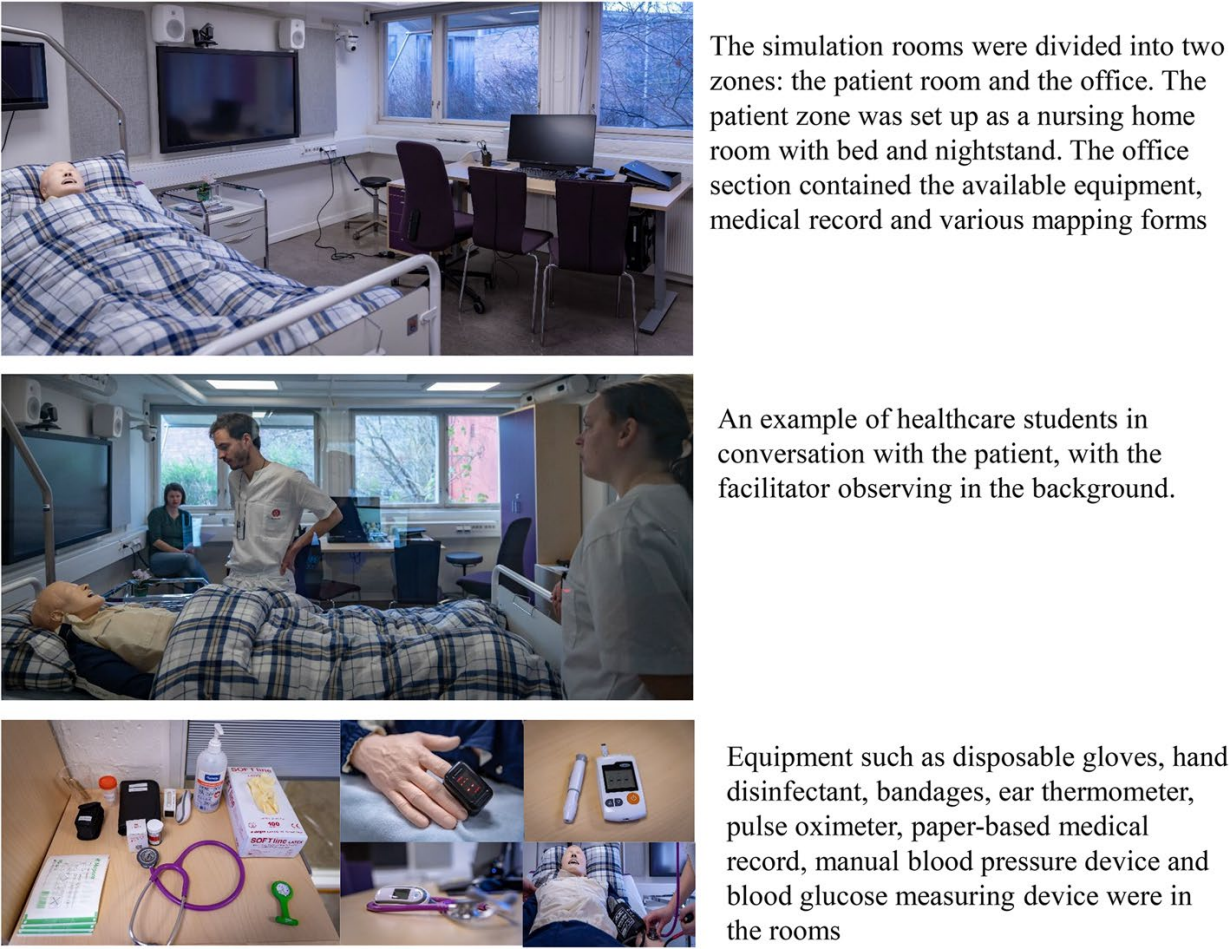
Simulation set-up and available equipment

The simulations took place in a research laboratory at the University of Oslo. The simulation units were set up like rooms in nursing homes. The scenarios were created in collaboration with primary care health professionals and comprised common medical conditions from primary care: an older patient convalescing at a nursing home following surgery for a hip fracture. The patient then developed symptoms of either a urinary tract infection or pneumonia.

The students participated in both scenarios described in Additional file 2 during the simulation-based training. Two scenarios were conducted during each simulation-based training activity each preceded by a briefing and immediately followed by a debriefing [20]. The briefing provided an introduction to the simulation room, the available (technical) equipment and the patient simulator SimMan® by Laerdal Medical [21], as well as a reminder about confidentiality and an introduction to the scenario [22]. During the simulation, facilitators acted as the patient’s voice and answered the questions directed towards the patient. We instructed the students to act according to their distinct professional roles and future responsibilities. Each scenario lasted approximately 30 min. The debriefing took place directly after each scenario and lasted on average 25 min [23].

### Participants

We recruited medical students, master’s students in advanced geriatric nursing and bachelor’s students in nursing through purposeful sampling. Educational leaders in universities in central Eastern Norway facilitated the recruitment. The inclusion criteria were healthcare students in the final semester of their last or second-to-last year of education because they had completed most of their clinical practice rotation and thus presumably would have skills competence sufficient to be capable of participating in IPC. Potential participants that met our inclusion criteria received information about the study from contact persons at the different universities. The lead author LL was also invited to several lectures to talk about the project to recruit participants. A total of 27 healthcare students agreed to participate, ranging from 21 to 49 years of age (mean 31), and 21 were female and six were male. All participants in the simulation training took part in the FG interviews. Table 1 presents the details regarding the participants.

**Table 1:** Description of the participants

	*N* (%)	Prior participation in simulation		Prior participation in interprofessional simulation	
		Yes, *N* (%)	No, *N* (%)	Yes, *N* (%)	No, *N* (%)
Total	27 (100)	22 (82)	5 (18)	7 (26)	20 (74)
Medical students (MS)	10 (37)	8 (80)	2 (20)	2 (20)	8 (80)
Master’s students in adv. geriatric nursing (AGN)^a^	8 (30)	6 (75)	2 (25)	2 (25)	6 (75)
Bachelor’s students in nursing (NS)	9 (33)	8 (88.9)	1 (11.1)	3 (33.3)	6 (66.7)

^a^The AGN students have a minimum of 2 years of clinical experience as staff nurses before entering into the master’s programme

To maintain the anonymity of the participants, gender and name were excluded from the transcripts, and abbreviations were used, as can be seen in Table 1. The participants were numbered in the order they appeared in the interviews (e.g. NS1). The FG interviews were numbered in the order they were conducted (e.g. FG1).

### Data collection

We conducted the FG interviews in April 2019, just after the students had finished the simulations and completed the ICCAS questionnaire, to avoid conflicts with study schedules. Each student was a member of one of 10 interprofessional teams during the simulations. Two teams participated in the simulation each day, and they joined the same FG, resulting in five FG interviews. The lead interviewers were members of the research group with experience in qualitative research and with doctoral degrees in nursing (AM) and medicine (AMB, EOR). Each FG interview lasted between 60 and 90 min and had five or six participants.

The interviewers observed the simulations from behind a one-way mirror in the control room and did not interact with the students during the simulation. The FG interviews were audio-recorded and were exported to a secure data storage facility at the University of Oslo, then transcribed verbatim by LL.

### Data analysis

The transcripts from the FG interviews were analysed by systematic text condensation, in a four-step process [24]. First, we read the transcripts independently to get an overview and total impression and to identify preliminary themes. Secondly, we collaboratively identified and sorted the meaning units into code groups. In the third step, we abstracted condensates from each code group. Finally, we created synthesised descriptions by reconceptualising the condensates and chose the quotes that would best represent the synthesised description (golden quotes). The initial steps were conducted by LL and AMB independently (step 1) and in collaboration (step 2). Then, LL drafted the first versions of condensates (step 3) and synthetisation (step 4) and translated the quotes into English. For each step of the analysis, the research group read the material independently, collaboratively discussed, modified themes, reviewed abstractions and syntheses until reaching a consensus.

We used the software NVivo12 to organise and structure the data. As the analysis progressed, we organised the material into tables. Table 2 shows an example of the analysis.

**Table 2:** Example of analysis

Step 1	Step 2	Step 3	Step 4
General impression and identification of preliminary themes	Identification and coding of meaning units (first person)	Construction of artificial quotations (condensates) summarising several meaning units (first person)	Syntheses of contents into main themes and sub-categories Choice of golden quotes (third person)
Very realistic and similar to practice	MS1 (FG1): “I am in nursing home practice now and had my first day yesterday. This could have been yesterday! And it could be tomorrow.” NS2 (FG2): “I think they were really good cases. Because it’s the type of patient you would actually meet.” MS9 (FG5): “I especially think about the fact that it was so relevant. The topics were important, and the situations ones that you would often experience.” AGN6 (FG4): “And I also think that it was very good that we were told immediately that this is a nursing home and this is the available equipment in the nursing home, and that the doctor is present one day a week. This made it realistic.” MS7 (FG4): “You need those surroundings to make it is as believable as possible.”	I am in practice at a nursing home and this could have been yesterday, or tomorrow. It felt very realistic and relevant for primary care. This is also the kind of patient you would typically meet in healthcare. We were told immediately that this is a nursing home and what equipment we had access to. Having been in a nursing home, the resources and their availability felt realistic. You need to have surroundings that feel realistic to make the simulation believable.	**Main theme:** realism **Sub category:** recognition of realistic scenario and setting The students recognised the scenarios as realistic, the situations as authentic situations and ones that they would likely encounter in healthcare, and specifically in primary care. The students also described the setting in a nursing home as recognisable and realistic. They highlighted the necessity to have realistic surroundings that would make the simulation authentic. Golden quotes: MS9 (FG5): “I especially think about the fact that it was so relevant. The topics were important, and the situations ones that you would often experience.” AGN6 (FG4): “And I also think that it was very good that we were told immediately that this is a nursing home and this is the available equipment in the nursing home, and that the doctor is present one day a week. This made it realistic.”

The ICCAS questionnaires were analysed using IBM SPSS Statistics Version 27. ICCAS comprised the interprofessional competency communication, collaboration, patient- and family-centred care, roles and responsibilities and conflict management. Since prior validation studies recommend analysing ICCAS at an overall level to address change in interprofessional competence [17, 18], we used paired *t*-test to determine the difference in perceived abilities in the mean overall pre- and post-score (range 1–5). We analysed the differences in terms of Cohen *d* standardised effect size (“large” = values of ≥ 0.8, “moderate” = values between 0.79 and 0.50 and “small” values between 0.2 and 0.49) and 95% confidence limits [25].

#### Strategies to enhance trustworthiness in the analysis

The authors are nurses (LL, AM), medical doctors (RBJ, EOR, AMB) and an educator (BM). Collectively, our experience combines primary care and medical educations, as well as research, teaching, curriculum planning, workplace learning and simulation-based training. Our backgrounds might have influenced our preunderstanding of the simulation setting, the scenarios and the students’ experiences. However, having co-authors with different, yet complementary backgrounds might also help in ensuring the legitimacy of our interpretations [26]. By reporting the process of analysis and providing examples of codes, construction of condensates, syntheses and themes in Table 2, we have brought a certain transparency to the process. Through the research group’s collective reading and analysis, we have worked to enhance the trustworthiness of the results [26].

## Results

Three main themes emerged from the analysis of the FG interviews: *realism*, *uncertainty* and *reflection*. Within *uncertainty*, the sub-themes “unspecific situations”, “time to collaborate” and “room for communication” became apparent. In *reflection*, the sub-themes “opportunities not present in practice”, “developing confidence” and “better prepared for the future” emerged.

### Realism of the scenario

The students recognised the scenarios as realistic, authentic and likely to be encountered in healthcare and, specifically, in primary care.

*MS9 (FG5): I especially think about the fact that it was so relevant. The topics were important, and the situations ones that you would often experience.*

Furthermore, the students described the nursing home setting as recognisable and representative. They noted that to make the simulations authentic, you needed to have such surroundings. The students seemed to manage to conceptualise the scenario in a clearer way based on the information provided and the environmental set-up.

*AGN6 (FG4): And I also think that it was very good that we were told immediately that this is a nursing home and this is the available equipment in the nursing home, and the doctor is present one day a week. This made it realistic.*

As such, the students emphasised that it was important to have authentic, recognisable scenarios and that having the setting and equipment described beforehand allowed them to better envision the scenario. Together, these statements illustrated that including information about setting, surroundings and available equipment as well as scenario description in the pre-briefing was important to prepare the students for the simulation.

### Uncertainty

#### Unspecific situation

The students expressed that in prior simulation experiences, they were usually provided with predefined ways of solving the problem, either through algorithms or checklists. In these scenarios, however, they experienced an ambiguous situation, where the right solution did not clearly stand out. They described it as they knew something was going on, but the unspecific clinical signs made the situation difficult to grasp and therefore difficult to analyse.

*AGN3 (FG2): Very often it starts with the fact that you realise that there is something going on. Without having anything specific, everything is a bit vague. That’s what it’s often like.*

The realisation that the patient’s situation was changing encouraged the students to pay careful attention to the vague and undefinable signs that were found in the clinical examination. The students explained that especially with elderly patients, the clinical signs might not be as apparent or lead to textbook solutions. The presentation of vague and unspecific symptoms made the students think more broadly in their clinical assessments, as a symptom could be interpreted in several ways. Consequently, they were less certain of the patient’s diagnosis.

*MS6 (FG3): Because there were vague symptoms, you had to think a bit more broadly. You think there can always be something more to it. And, that this kind of assessment feels a little unfamiliar.*

The students expressed that they were unaccustomed to these assessments, especially because there was no quick fix or easy solution. However, the students perceived this experience as positive because, in nursing homes, and primary care in general, they would often experience vague clinical situations. As such, it seemed that the scenarios were recognised as important learning activities to prepare for real-life situations. The use of sub-acute scenarios shifted their focus to the inherently complex health services that are provided in primary care on a daily basis and appeared to renew the students’ understanding of the many different challenges that can occur.

#### Time to collaborate

The students emphasised that having an adequate time to practice scenarios together in a calm setting offered the opportunity to ask additional questions, listen to one another and engage in group discussions to solve problems.

*MS4 (FG2): When you have so much time and it is quite calm, you have the opportunity to listen, and to ask, “What do you think? Is there anything we have not thought of?”*

Although there was enough time to work on it, the clinical problem itself was less clear-cut. The students reported that they could not take any shortcuts because the symptoms were so vague. They had to discuss what they were unsure of and do a full clinical examination.

*AGN3 (FG2): If it is a cardiac arrest, pretty much everyone knows what to do, and you cooperate. But when it is so vague, you get a discussion of everyone’s knowledge, and it’s completely different. You get to use each other’s competence in a completely different way than if it was a very specific and dramatic situation.*

In contrast, the students found that a simulation solving an acute care situation such as a cardiac arrest where they follow a predefined algorithm was more rehearsed and explicit as they would know what to do and how to react. Sub-acute scenarios provided the students with an opportunity to use each other’s competences in new ways. The vagueness, the students said, consequently led to another kind of insight of what the other students knew and how they could contribute, as they had to share their knowledge to expand on the problem. The simulation seemed to contribute to increased understanding of the competence the different educations provided, and how they could complement each other. Thus, when they combined their different perspectives, it helped reduce the uncertainty. This indicated that adding different professional perspectives enhanced the joint discussion and thus increased their learning outcome.

#### Room for communication

Based on their prior experience with simulation, the students said they expected an extraordinary situation even though they were prepared for sub-acute scenarios. The fact that the clinical condition in the scenario did not overwhelm them was highlighted as positive. Thus, it was possible to focus on the team’s interactions and communication, which they deemed important.

*MS5 (FG3): When it’s not medically precarious and acute, you get a little more time to actually communicate. And that’s what’s most important.*

The students appreciated that the clinical condition did not decline rapidly, as it gave them more time to react and collaborate. They pointed out that in a medically complicated scenario, you could just as well end up with a situation where one team member dominates.

*NS10 (FG5): If it gets too complicated and there’s a dispute between the professions, and the one who speaks loudest overrides the rest of the group. Some just cave in and heed to the one who has the strongest opinions.*

As the students pointed out, complicated cases could negatively affect the communication and collaboration.

### Reflection

#### Opportunities not present in practice

Several students talked about the simulation as being similar to practice and yet not so, especially regarding time to reflect during the simulation and in the debriefing. They highlighted that in these scenarios, they had time to talk through the clinical picture of the patient together and really listen to each other. In real-life practice settings, they said it might be busy and chaotic, and opportunities for reflecting together and share profession-specific knowledge about the patient were less available.

*AGN3 (FG2): I learned a lot from seeing what the others reflected on. Here you do the reflection together. You see what the different students see; there is not always room for that when you work.*

The students also emphasised that having the opportunity to sit down together in the debriefing and reflect on what they did enhanced the learning outcome. In the debriefing, the students appreciated the possibility to talk about how they communicated and collaborated in the simulation sessions individually and as a team. They particularly pointed out that they valued the focus on raising awareness and understanding of the situation together without merely pointing out what went wrong.

*MS10 (FG5): I absolutely believe that training in controlled settings where you get time to reflect afterwards has great value that is difficult to include in practice. Because in practice, you are dependent on a supervisor taking time to include reflection and a department with suitable conditions for reflection with others*

The students explained that it was not always possible to take an active part in collaboration in clinical practice, and the possibility to interact with other students or healthcare personnel could be limited or non-existent. In clinical practice, they experienced that there was little time given over to reflect together with others. This appeared to illustrate that profession-specific learning goals in clinical practice are still the most common and that interprofessional activities where the students have time to reflect with others are scarce.

*NS9 (FG5): We know that, in practice, we can call the priest, social worker, or nutritionist and get them up there and then talk to them. But you may not know how you would collaborate with them in that meeting. You are doing that in here. What we do here is very important in shedding light on how we should collaborate.*

By sharing experiences and reflecting together, the students indicated that they got to know the competences of the other healthcare professions first hand. This was perceived as important for managing collaboration. The students described the simulation setting as a good way to become more aware of the roles and responsibility they would assume in their future work life. It also gave room to reflect on how to collaboratively solve problems, not just on the idea that collaboration was necessary. Experiencing the benefits of IPC may also lead to enhanced respect for each other’s profession. As such, the students voiced the importance of participating in training that enhances the quality of IPC.

#### Developing confidence

The students said that participating in the simulation made them more aware of themselves for better or worse, in terms of how they behaved and dealt with situations. They described the experience as discovering themselves in a new way. Consequently, the experience appeared to develop their confidence to engage more actively in IPC.

*AGN4 (FG3): With simulation, I see that if I can talk to the medical student, then maybe I can talk to a real doctor. You see proof that it’s actually possible to talk to other professional groups.*

The students found that as the simulation progressed, they got more comfortable with expressing their opinion with the team, which made it possible to have a clinical conversation across professions. Solving the scenario, they explained, provided an opportunity for participating in discussions in a safe environment as equals. The creation of a safe environment allowed the students to dare to present their perspectives and express their opinions.

*NS5 (FG3): I learned today that I don’t have to be afraid. If I have some knowledge or something that I think of, with the patient in mind, I will just say it.*

The students explained that when they discussed together, they realised that they had an important role to play. Thus, the joint problem-solving activities the scenarios provided seemed to increase their experience of themselves as important contributors to the interprofessional discussion. Consequently, the simulation experience led to newfound confidence in the students’ abilities to participate and voice their opinions. This confidence appeared to reassure the students in their own role as healthcare professionals. When reassured in their own role, they managed to benefit from the others’ competence and mutually create joint knowledge.

#### Better prepared for the future

The students indicated that the experiences from the scenarios would be long lasting because the simulation created practical memories they could recall later.

*MS5 (FG3): It’s the kind of experience that you can come back to and reflect on. You can call on it in different settings and think, “Oh, yes, we did this that time.”*

The students said that taking part in the simulations would help them deal with similar situations in the future. Facing such issues in a safe environment during education gave the students a sense of assurance for future work.

*NS6 (FG4): If you could act through it in advance and be trained beforehand, you can handle it better later, in terms of how to talk to each other.*

Thus, the students reported that interprofessional collaboration could become something familiar and manageable because of prior training. Participating in the scenarios seemed to provide the students with a clearer frame of reference for problem-solving in future situations. Having useful experiences to refer could provide security since they had faced such issues during education. Consequently, this type of scenarios could prepare the students for future IPC.

### Self-reported interprofessional competence

In addition to the material from the FG interviews, all 27 participants completed the ICCAS questionnaire. The results from the ICCAS questionnaire showed that after participating in the scenarios, the students reported a positive change in self-assessed interprofessional competence. There was a statistically significant increase in the mean sum score from pre-scores (mean = 3.64, SD = 0.65) to post-scores (mean = 4.4, SD = 0.3), *t* (26) = 6.67, *p* < .001 (two-tailed). The mean difference, 0.76, 95% CI [0.53, 0.99], represented a large effect of *d* = 1.29.

## Discussion

In the findings reported here, the students emphasised the importance of authentic and recognisable scenarios. They described that the vague and unspecific symptoms in the scenarios created an uncertain situation where it was difficult to find a clear direction. The students repeatedly emphasised, however, that this experience was positive. They acknowledged, with some surprise, the complexity the sub-acute scenarios presented and the opportunity that arose for them to focus on collaboration and communication. Further, the students reported increased confidence and preparedness for future work. Our results from ICCAS also supported that participating in the scenarios led to a positive change in self-assessed interprofessional competence. Furthermore, we discuss the potential for the sub-acute scenarios to promote interprofessional collaborative learning opportunities for healthcare students.

### Collaborative problem solving in a realistic setting

An important finding from this study was the students’ positive response to the sub-acute scenarios, especially their seeing scenarios as authentic and realistic learning situations. The recognisable scenarios, together with information about the setting and available equipment, were important factors in getting students to engage in the simulation. Considering the simulation activity as a social practice where learning is constructed in interaction between the participants, environment and equipment, it highlights the importance of pre-briefing to create a safe and recognisable environment for the students to interact in [22, 27, 28]. Thus, they seemed to manage to utilise the resources available in the room and frame the simulated situations into something manageable.

This supports the findings showing that IPE has to be meaningful and relevant, with authentic activities, to be able to support interprofessional learning [4, 29]. Further, for a learning experience to be of value and to prepare the students for future teamwork, structured opportunities for active engagement need to be made available [11, 30]. Thus, IPC experiences involving engagement and opportunities to interact, rather than passive observation of teamwork, are found to have more impact on interprofessional learning and competence development [31–33].

The unspecific symptoms presented in the scenarios created an uncertain situation for the students, where the patient’s problem or diagnosis was unclear. As such, the sub-acute scenarios exposed the students to the complexity often presented by this patient group, where accurate diagnosis can be difficult due to atypical symptoms [34]. Since there was no detailed algorithm to follow, the outcome depended on the students’ capacity to discuss, identify signs and symptoms and use relevant knowledge to solve the patients’ main concerns. Students who actively share information, discuss and draw on one another’s resources and competencies seem to manage defining the patients’ concerns and prepare for future care in collaboration [35]. In our study, the students highlighted that the relaxed pace of the scenarios, combined with a reasonable amount of time to complete them, made it possible to focus on the interactions and communication within the team, to ask each other questions and discuss and reflect together without being overwhelmed. When students recognise the simulation-based activity as a safe environment, it can motivate them to perform at the edge of their expertise [22], which might enable them to expand on the learning activity and enhance their knowledge. In our scenarios, the students recognised the setting as a safe environment, which made them willing to ask questions, listen to reflections from others and contemplate on the best way forward together, although it might highlight skills deficiencies.

When developing scenarios for simulation-based training, careful consideration of the level of difficulty and complexity is necessary to optimise the learning opportunities [27, 28, 36]. It is important to take into account that the students participating in the scenarios are there to train on competence they have not yet fully acquired [37]. Thus, a mismatch between the difficulty and complexity of the scenario and the students’ capacity to make sense of the scenario could compromise the learning opportunities. As the students explained, complicated cases can breed poor communication, as one team member may dominate. As such, scenarios where the patient’s condition is stable seem to provide students with more time and opportunity to emphasise team collaboration [38, 39].

The students’ experiences of a collaborative learning potential in simulation seemed to come from the combination of a *realistic* scenario and a *practice space* for IPC in the simulations. For students to be prepared for the expected collaboration, educators have to create spaces to train for IPC in healthcare education [15]. The foundation for fruitful learning spaces have to be laid in the pre-briefing to get the students to engage in the simulation and interact with the participants, scenario and environment [22]. Without these spaces, it is difficult for healthcare students to get to know one another and find ways of working together [40]. The practice space for IPC in the sub-acute scenarios seems to provide the opportunity for healthcare students to explore one another’s perspectives and use one another’s competencies interprofessionally.

### Learning opportunities

Through IPE-based simulation training of sub-acute situations, this study shows that the following learning potentials can be realised: establishing greater confidence in handling uncertain, sub-acute situations through IPC, understanding their own and others’ perspectives and competencies and strengthened confidence in their own IPC competencies and contributions for future work. These practice spaces for IPC emerge during the joint examination of the clinical situation and is strengthened through reflection.

Reflecting on the simulation experience, especially the debriefing, is seen as a cornerstone in simulation-based training for students to reconstruct their experience into learning [27]. There are several ways of facilitating scenario debriefing [41, 42], making it important for educators to make well-considered choice of debriefing strategy beforehand. In this study, the facilitators were instructed to follow the debriefing framework proposed by Rudolph et al. [23] where the focus is enhancing awareness and understanding of the situation. The framework highlights creating a safe learning environment where the students feel comfortable discussing successes and failures to understand and learn of their actions. The students in our study appreciated that the facilitators did not solely focus on what went wrong, but prompted questions, thoughts and opinions that engaged the students to contribute actively with their own reflections and perspectives on collaboration and communication.

In our study, the realistic but vague and unspecific signs and symptoms in the scenarios without a clear conclusion created uncertainty that challenged the students’ competence, their role understanding and task sharing. However, the uncertainty also mobilised their resources as they resolved the uncertainty by communication and joint reflection in the simulation and during the debriefing. As such, the development of IPC competence took place both during the scenario and in the debriefing. The quality of the debriefing seems as important for the development of IPC competence as the quality of the scenario since the debriefing is where the participants shift their perspective from the action to the reflection on actions and common experiences from the scenario [23]. This study suggests that the scenarios allowed for discussion and joint reflection and that the simulation training may lead to enhanced understanding of one another’s sense of competence and scope of practice. Most especially, the simulation provided an opportunity for equal discussions in a safe environment. This supports the findings suggesting that feeling safe in a learning situation fosters confidence in one’s role and willingness to participate in a team [8, 9, 12, 14]. Moreover, our results may indicate that the scenarios provided safe ways of developing interprofessional collaborative competence where different perspectives are valued. Unequal power relations and hierarchical structures are seen as barriers for learning [43, 44]. We highlighted that everyone’s knowledge and perspective were necessary to solve the problem which seemed to promote a non-hierarchical learning environment and strengthen the students’ confidence in their interprofessional competence.

The students said that though similar to clinical practice, the simulated setting was also different. The joint discussions and reflections about the patients’ clinical picture they experienced during the simulation session were not usually encountered in clinical practice or work, neither was the structured debriefing. This might be seen as an educational paradox, in which students participate in IPE to prepare for future interprofessional practice that rarely takes place. Thus, it can be challenging to prepare students with IPC competencies if they do not find opportunities to practice in clinical work. Consequently, those students might not consider IPC as important in real-life clinical work [5]. At the same time, education institutions have a responsibility to include high-quality IPE, and thereby contribute to the quality of IPC in the future. Otherwise, newly graduated students risk entering their professions without the interprofessional collaborative competence needed to work efficiently in future healthcare teams [2].

The simulation-based experience offered a frame of reference for future problem solving. Thus, it seems that the realistic setting not only enhances learning, but also makes it more transferable for future situations, confirming existing research which suggests that authentic, interactive and competence-building IPE experiences create lasting impressions [29, 30]. However, it is important to highlight that realism—or fidelity—does not mean that everything must be as found in practice, without exception. Simulation fidelity relates to the educational value of the simulation which means that the necessary level of realism should be evaluated to create the required learning environment [28]. In our study, we have shown that the students valued the authentic and realistic scenarios. Although the simulation was conducted in a simulation centre with a SimMan as the older nursing home patient, the student perceived the situation as realistic due to the authentic scenario description, convincing access to equipment, presentation of vague clinical signs and credible information provided in the medical record. This highlights that to create a realistic simulation experience—or the right amount of fidelity—it has to contain physical elements but also situations the students manage to make sense of and experience as relevant [28, 39]. In our study, albeit the fact that not everything was identical to practice, the abovementioned factors contributed to create a context where the students experienced a sense of recognisability and, thus, engaged in the scenarios.

Systematic IPE could be an advantage for future teamwork, as the students explained that having experienced IPC, they felt prepared to contribute in future IPC situations. The positive change in the students’ self-reported competence score supported that participating in the scenarios prepared the students for collaborative practice. Other studies have also found that IPC training develops competence and enhances the ability to engage in future interprofessional teamwork in clinical practice [33]. Exposing healthcare students to IPE during education can result in more graduates with IPC competence, which in turn can promote a positive change towards further interprofessional collaborative healthcare practice. Thus, the IPC learning outcomes the students achieved in these scenarios could be transferable to other settings and situations.

### Strengths and limitations

A strength of this study is that it expands simulation-based IPE as a strategy to prepare healthcare students for future IPC and shows the potential of adding simulations of sub-acute primary care scenarios to IPE. We acknowledge that the participating students might be more positive about simulation and IPC than other students might. Reasons for non-participation were, however, mainly the lack of time and not getting time off from work or clinical practice. The simulations and FG interviews were conducted in 1 day to facilitate participation and avoid study schedule conflicts. We do not know if the students would have shared the same viewpoints in the FG interview had they had time to process the experience over a longer period. However, FG interviews provided us with detailed and rich descriptions of the students’ immediate experiences. An interesting follow-up study could be to investigate how the students experienced the simulation after having entered healthcare as healthcare professionals. Although ICCAS added the students’ individual and anonymous assessment of their own competence, we have too small a sample size to evaluate the effect of the sub-acute scenarios. We performed the FG interviews with the whole interprofessional group and not divided by professions. This might have inhibited some participants to speak freely, since they might be influenced by how they think they are expected to act in their future professional roles. However, since the students were in these groups for 1 day only, we consider it unlikely that this was a major problem. The analysis was based on the researchers’ interpretations of the transcripts. The students have not had the opportunity to comment on our interpretations, and we acknowledge that their interpretations or explanations of the transcripts may differ from ours. Although research promotes the use of simulation to support IPE, there are few studies with sub-acute scenarios from primary care. Thus, our study contributes to a new perspective on how to facilitate for IPE in healthcare education. These scenarios seem to be feasible for implementation in healthcare education. Adding observers with specific tasks related to observation of the simulation activity could be one way to scale up to accommodate real student numbers and consequently avoid inactive participants in the scenarios. Then, the students could take turn in taking part in a scenario and observing their peers taking part in another scenario. The scenarios also have potential to be expanded to include other healthcare professions, which would have been an interesting opportunity for further study.

## Conclusions

The present study shows that simulation-based IPE with sub-acute primary care scenarios in healthcare education contributes to the development of the collaborative competence. The students valued the authentic scenarios and expressed that solving the scenarios increased their competence in IPC and prepared them for future work. The sub-acute scenarios, although complex in relation to the unspecific and vague symptoms, promoted collaborative learning opportunities for the students due to the authenticity and sufficient time to discuss and reflect. Introducing simulation-based IPE with a focus on primary care scenarios can supplement more common acute care simulation approaches for developing the collaborative competence required to work in healthcare teams.

## Supplementary Information

**Additional file 1.**

**Additional file 2.**

## Data Availability

The datasets generated and/or analysed during the current study are not publicly available due to the data corpus still being subject to analysis but are available from the corresponding author on reasonable request. Additional file 1: Interview guide; Additional file 2: Scenario description.

## Abbreviations

IPE: Interprofessional education
IPC: Interprofessional collaboration
MS: Medical student
AGN: Master’s students in advanced geriatric nursing
NS: Bachelor’s students in nursing
FG: Focus group
