# Supplementary material for: A preliminary simulation-based qualitative study of healthcare students’ experiences of interprofessional primary care scenarios
Source: Adv Simul (Lond). 2022 Mar 21;7:9. doi: 10.1186/s41077-022-00204-5 (PMC8935844; doi:10.1186/s41077-022-00204-5)
Supplement: Supplementary file 1 — Additional file 1. [file 41077_2022_204_MOESM1_ESM.docx]

**Guide for focus group with medical students, advanced geriatric nursing students and nursing students**

**Presenting the aim of the focus group interview:**

The aim of a focus group interview is to gain insights, thoughts, experiences and beliefs of a situation, in this case the simulation scenarios. It is not an aim to gain consensus on the themes under discussion, but we would like to hear about your experiences. Feel free to elaborate on one another’s statements.

**Opening questions (asking each student):**

- We would like you to introduce yourself and tell a little about your previous experiences with simulation in general and interprofessional simulation specifically
- If participated in any kind of simulation, if it was at school or in practice

**Key themes and probing questions**

Theme 1: The scenarios

What are your thoughts on these scenarios?

- Probing questions:
  - How was it to go through a scenario in a normal nursing home setting?
  - Was something missing?
  - Was there anything that was particularly good?
  - What was your role in the scenario?
  - How did you clarify tasks and roles in the team?
  - What could you bring with you from the first scenario to the second? Do you have examples?
  - Did it make sense to carry out the simulation twice? Should the two scenarios have been more different?

Theme 2: Interprofessional collaboration and simulation

What are your thoughts about having interprofessional simulation training during education?

- Probing questions:
  - Can you describe what interprofessional collaboration means to you?
  - How do you view your own role in interprofessional collaboration?
  - What kind of experience do you have with what other professions or healthcare students learn during their education?
  - If you were to create scenarios focusing on collaboration between students, what would you have focused on?
  - What does it take to create credible and realistic scenarios for training of collaboration?
  - What should the scenarios focus on to be especially relevant for primary care?
  - What are the advantages or disadvantages of conducting sub-acute interprofessional simulation scenarios?
  - What do you think about having this as a compulsory part of the education?

**Summary and ending questions**

The moderator sums up

Is there anything else you would like to say, elaborate on, clarify etc.? Anything you have forgotten to say or want to emphasize.
