# Supplementary material for: A preliminary simulation-based qualitative study of healthcare students’ experiences of interprofessional primary care scenarios
Source: Adv Simul (Lond). 2022 Mar 21;7:9. doi: 10.1186/s41077-022-00204-5 (PMC8935844; doi:10.1186/s41077-022-00204-5)
Supplement: Supplementary file 2 — Additional file 2. [file 41077_2022_204_MOESM2_ESM.docx]

**Description of the scenarios**

Scenario briefing:

You will shortly be presented with a patient case where a patient is staying at a nursing home. You are all at work at the nursing home today.

Firstly, we will go through the tasks in the simulation. You should perform a clinical assessment, agree on a reasonable clinical problem or diagnosis, and develop a shared treatment plan together during the simulation. We expect you to act according to your future professional role in the scenario. You have 30 minutes to perform the tasks.

A week earlier, an old man fell at home and broke his right hip. He went to hospital for surgery. On the fourth post-operative day, he was transferred to the rehabilitation ward at the nursing home. A bladder catheter was removed before the patient left the hospital.

In the nursing home, training is in progress, but he is tired and in pain. The patient trains with a physiotherapist daily, otherwise spends most of his time in bed. He is not sleeping well.

The medical doctor is present in the nursing home once a week.

Medical history found in the medical record:

- Male, 80 years old
- Pensioned accountant living alone in a house with two floors
- Wife died a year ago with moderate Alzheimer. One son.
- No cognitive decline
- Heart failure and hypertension, both stable with medication
- Transient ischemic attack (TIA) in 2015, followed by secondary prophylactic treatment

Additional resources in the medical record

- List of medications
- Admission papers to the nursing home
- Day to day nursing documentation at the nursing home
- Discharge papers from orthopaedic doctor, nurses and physiotherapists at the hospital
- A copy of the medication list from the hospital

The urinary tract scenario:

The patient had an accident with urine on his way to the bathroom, and needed help with his trousers. You are asked to go to the patient to assess the situation.

Main clinical signs:

- No airway obstruction
- Normal respiratory rate and normal breath sounds
- Normal heart sounds
- Blood pressure 118/80, pulse 92
- Temperature 37,7 Celsius
- Frequent urination, cloudy and dark.
- Urinary dipstick: Positive for leukocytes, blood and nitrites
- Bladder scan: 375 ml before urination, 175 ml after urination
- No upper back and side (flank) pain
- No sign of infection in the operation wound on the right hip
- CRP (C-reactive protein): 45

The pneumonia scenario:

The patient had trouble finding his room after dinner. He appeared slightly agitated during dinner. You are asked to go to the patient to assess the situation.

Main clinical signs:

- No airway obstruction
- Normal heart sounds
- Elevated respiratory rate of 18
- Late inspiratory crackles on the right lung, normal left lung
- Dry cough at night, slight dyspnoea when talking
- Blood pressure 122/80, pulse 90
- Temperature 37,9 Celsius
- Normal urination
- No sign of infection in the operation wound on the right hip
- CRP (C-reactive protein): 50
